# A Multispecies Fungal Biofilm Approach to Enhance the Celluloyltic Efficiency of Membrane Reactors for Consolidated Bioprocessing of Plant Biomass

**DOI:** 10.3389/fmicb.2017.01930

**Published:** 2017-10-10

**Authors:** Charilaos Xiros, Michael H. Studer

**Affiliations:** Laboratory for Bioenergy and Biochemicals, School of Agricultural, Forest and Food Sciences, Bern University of Applied Sciences, Bern, Switzerland

**Keywords:** multispecies biofilm membrane reactors, *Trichoderma reesei*, *Aspergillus phoenicis*, lignocellulose, cellulases, *β*-glucosidase, fungal biofilms

## Abstract

The constraints and advantages in cellulolytic enzymes production by fungal biofilms for a consolidated bioconversion process were investigated during this study. The biofilm cultivations were carried out in reactors designed for consolidated bioprocessing Multispecies Biofilm Membrane reactors, (MBM) where an aerobic fungal biofilm produces the lignocellulolytic enzymes while a fermenting microorganism forms the fermentation product at anaerobic conditions. It was shown that although mycelial growth was limited in the MBM reactors compared to submerged cultivations, the secretion of cellulolytic enzymes per cell dry weight was higher. When *Trichoderma reesei* was used as the sole enzyme producer, cellobiose accumulated in the liquid medium as the result of the deficiency of *β*-glucosidase in the fungal secretome. To enhance *β*-glucosidase activity, *T. reesei* was co-cultivated with *A. phoenicis* which is a *β*-glucosidase overproducer. The two fungi formed a multispecies biofilm which produced a balanced cellulolytic cocktail for the saccharification of plant biomass. The mixed biofilm reached a 2.5 fold increase in *β*-glucosidase production, compared to the single *T. reesei* biofilm. The enzymatic systems of single and mixed biofilms were evaluated regarding their efficiency on cellulosic substrates degradation. Washed solids from steam pretreated beechwood, as well as microcrystalline cellulose were used as the substrates. The enzymatic system of the multispecies biofilm released four times more glucose than the enzymatic system of *T. reesei* alone from both substrates and hydrolyzed 78 and 60% of the cellulose content of washed solids from beechwood and microcrystalline cellulose, respectively.

## Introduction

Biological decay, bioremediation and biodegradation of plant biomass are performed by the combined action of diverse microorganisms or by complex microbial communities. A paradigm of cellulolytic multispecies biofilms from nature is the cattle rumen where cellulose degradation is mainly performed by such multispecies biofilms which are formed on the lignocellulosic particles and on the rumen walls (McAllister et al., 1994). The successful degradation of cellulose by these microbes is based on the dynamic equilibrium among the different species: Rumen microorganisms benefit from each other's metabolism, in order to effectively access and consume their preferred substrates (Prescott et al., 2002; Nagaraja, 2016). These interactions create distinguished ecological niches, making the rumen suitable for many different microorganisms.

Microbial biofilms and microbial consortia have been used in various bioconversion processes of lignocellulosic biomass (Villena and Gutierrez-Correa, 2007; Wang and Chen, 2009; Kalyani et al., 2013). The formation of multispecies biofilm structures is beneficial for the bioconversion when the involved microorganisms require different conditions and/or when vicinity satisfies their (not always mutual) needs. Wang and Chen foresaw the possibility of a highly consolidated bioprocess (HCBP), which would incorporate delignification, saccharification and sugars co-fermentation in a single reactor inhabited by a multispecies biofilm (Wang and Chen, 2009). The consolidated bioconversion bioprocess integrates as many conversion steps as possible in one module and/or in one microbial host. Many different approaches of consolidated bioprocessing have been developed over the last decades, using different cultivation techniques, mainly in the framework of bio-ethanol research projects. The aim was to make the production of lignocellulosic ethanol economically attractive, by decreasing the cost of enzymes production and transport as well as the number of unit operations (Jouzani and Taherzadeh, 2015).

Brethauer and Studer (2014) developed such a CBP reactor for direct conversion of plant biomass to liquid fuels and chemicals, named Multispecies Biofilm Membrane (MBM) reactor. In this system, the simultaneous achievement and separation of aerobic and anaerobic conditions in the reactor allows fungal cellulolytic enzyme production and alcoholic yeast fermentation of the hydrolysis-derived sugars at the same time. After inoculation, a fungal biofilm is formed on a membrane, as the oxygen required for fungal growth and lignocellulolytic enzyme production is introduced to the cultivation only by diffusion through this membrane. Therefore, the fungal cells attach to the membrane; they grow and form a biofilm on it. The fungal biofilm itself is an oxygen sink, creating anaerobic conditions on the side of the liquid medium where the fermenting microorganism(s) may form an anaerobic biofilm or grow in the liquid, as the released fungal enzymes degrade lignocellulose to monomeric sugars (Brethauer and Studer, 2014).

The enzymatic hydrolysis of cellulose and hemicellulose is the slow step of most biomass bioconversion processes (Revin et al., 2016). Various enzymatic activities should act in synergy to degrade these polymers to free sugars, which will then be converted to the final products by the fermenting microorganisms. Cellobiohydrolases (CBHs), *β*-Glucosidases (BGs), endoglucanases (EGs), lytic polysaccharide monooxygenases (LPMOs) cleave different bonds on the cellulosic molecule. The lack of one of those slows down the hydrolytic process not only due to inability of the enzymatic system to cleave certain bonds, but also because the accumulation of intermediate products has inhibitory effects on the enzymatic activities. The presence of *β*-Glucosidase activity in sufficient quantities in the enzymatic cocktail is crucial in order to efficiently degrade cellobiose, which is a strong inhibitor of most cellulolytic enzyme activities (Philippidis et al., 1993; Singhania et al., 2013). Solid state cultivation (SSC) has been widely used for lignocellulolytic enzymes production, based on the well-known ability of fungi to grow on solid surfaces in nature. SSC studies on *Aspergillus phoenicis* which is a *β*-Glucosidase over producer have shown that co-cultures with other cellulolytic fungi enhance the cellulolytic efficiency of the produced enzymatic cocktail. SSC co-cultivations with *Trichoderma reesei* produced a very balanced enzymatic profile and showed about four times increase in *β*-Glucosidase production, in comparison with *T. reesei* alone (Wen et al., 2005).

The surface adhesion ability of fungi has been applied in biofilm fermentations which differ from SSC regarding the quantity of liquid being abundant (and free) in the culture environment (Gutiérrez-Correa et al., 2012). Fungal biofilms have been studied regarding their biotechnological applications (Villena and Gutierrez-Correa, 2007; Wang and Chen, 2009; Gamarra et al., 2010; Gutiérrez-Correa et al., 2012; Borghi et al., 2015) including lignocellulolytic enzyme production by *Aspergillus* strains (Villena and Gutierrez-Correa, 2007, 2012; Gamarra et al., 2010). *A. phoenicis* is known to be able to form biofilms in certain conditions; however its lignocellulolytic enzymes production, as well as biomass conversion applications of this fungus have not been studied in such cultivations.

In this study we evaluated the fungal biofilms as enzyme producing cultivations in comparison with the submerged cultivations. During *T. reesei* cultivations, the accumulation of cellobiose due to the lack of *β*-Glucosidase was shown. It was demonstrated that the MBM process can host more than one aerobic fungus in order to maximize the cellulolytic efficiency of the system. The cellulolytic efficiency of a single fungus biofilm (*T. reesei*) was compared with the multispecies biofilm (*A. phoenicis* and *T. reesei*) in the MBM system. *A. phoenicis* was selected, based on its secretome profile and its optimal growth conditions. We evaluated and compared the produced enzymatic extracts during enzymatic hydrolysis reactions using Avicel and washed solids from hydrothermally pretreated beechwood.

## Materials and methods

### Strains and chemicals

*T. reesei* RUT C 30 *and A. phoenicis*, both acquired from VTT culture collection (VTT Numbers: D-86271and D-76019, respectively) were used in this study. The fungi were received in lyophilized form and were reactivated according to the instructions by the provider. The fungi were grown on potato-dextrose-agar (PDA) slants for 5 days at 28°C. The slants were maintained as stock cultures at 4°C and renewed every 60 days. All chemicals were purchased from Sigma, Switzerland and VWR, Switzerland.

### Pretreatment, composition, and preparation of beech wood material for enzymatic hydrolysis

Beech wood (*Fagus sylvatica*) chips were milled to a final particle size below 1.5 mm. Steam explosion pretreatment was applied. A custom-built steam gun system (Industrieanlagen Planungsgesellschaft m.b.H., Austria) was used (Pielhop et al., 2016). Two hundred and fifty gram of wood with a moisture content of 6% w/w were inserted into the reactor chamber and saturated steam was injected to pretreat the material. Various experiments on beech wood steam explosion pretreatment revealed that different pretreatment conditions lead to an optimal xylan yield (180°C for 24.8 min) and to a maximum glucan yield (230°C for 14.9 min). Thus, beech wood were pretreated at 230°C for 14.9 min. After pretreatment, the slurry was collected, filtered and washed three times with an overall volume of de-ionized H_2_O equal to three times the volume of the slurry. The washed solids were then dried at 55°C until constant weight. The dry matter content was measured using a moisture analyzer (Mettler Toledo HB 43 -S, Switzerland). The cellulose and lignin contents in the pretreated beech wood washed solids (PBWS) were estimated using the NREL protocol for the determination of Structural Carbohydrates and Lignin in Biomass (Sluiter et al., 2008). The material was grounded in a Ball mill (Retsch MM 400, Haan, Germany) before analysis. Analyses were performed in triplicate. The same material was used in hydrolysis reactions.

### Inoculum preparation

For inoculum preparation, 6 mL deionized sterile water, containing 1% v/v Tween 80, were added to a PDA slant of the stock culture and aliquots (2 mL) of the mixture were used to inoculate 250 mL Erlenmeyer flasks (sterilized at 121°C for 20 min and cooled prior to inoculation) containing 50 mL of Mandels medium (containing the following ingredients (in g L^−1^): KH_2_PO_4_, 2; (NH_4_)_2_SO_4_, 1.4; MgSO_4_·7H_2_O, 0.3; CaCl_2_·6H_2_O, 0.4; urea, 0.3; peptone, 0.75; yeast extract, 0.25; and 1 mL L^−1^ trace element stock). The trace element stock contained (in g · L^−1^): FeSO_4_·7H_2_O, 5; MnSO_4_·H_2_O, 1.6; ZnSO_4_·7H_2_O, 1.4; CoCl_2_·6 H_2_O, 3.7 and 10 mL · L^−1^ concentrated hydrochloric acid and was sterile filtered. To avoid precipitation, 100 × CaCl_2_ and 100 × MgSO_4_ solutions were autoclaved separately). Avicel and glucose were used as the carbon sources during inoculum preparations for *T. reesei* and *A. phoenicis*, respectively.

### Cultivations in MBM reactors

Biofilm cultivations were performed in 32 mL MBM reactors set up as previously described (Brethauer and Studer, 2014). The reactors were filled with 31.5 mL of liquid medium. Avicel and washed solids from pretreated beech wood were used as the carbon sources at 2% w/w DM loading. The reactors were inoculated with 1.5 mL (~5% v/v) fungal inoculum (Brethauer and Studer, 2014) and incubated at 27°C. Wherever a co-cultivation of two fungi was performed, the second fungus was inoculated 48 h after the first inoculation (unless otherwise stated). Just before the second inoculation, the reactor was left to settle without stirring for 5 min and 1.5 mL of liquid (equal to the volume of the second inoculum) was removed from the reactor. All cultivations were performed in duplicate. Sampling was performed by harvesting a whole reactor.

### Sampling and fractionation of MBM cultivations

#### Liquid fraction and solid residue

After harvesting, the liquid fraction (including the residual solid substrate) was centrifuged at 20,000×g, at 4°C (Scanspeed 1580 R, Labogene, Denmark). An aliquot from the clear supernatant was used for the determination of enzymatic activities in the liquid fraction. The residue was washed as following: 10 mL citrate buffer pH 5, 50 mM, supplemented with 0.5% v/v Tween 80 were added to the pellet and the suspension was vortexed at room temperature for 1 h. The suspension was then centrifuged at 20,000×g, at 4°C. The washing procedure was performed twice. The two supernatants were pooled and were used for the determination of the enzymatic activities adsorbed on the solid substrate. The pellet after the second centrifugation was washed with 15 mL deionized H_2_O and checked for existence of fungal cells. A schematic representation is provided in Supplementary Figure 2, explaining the sampling and culture fractionation in the MBM system.

#### Biofilm fraction

The fungal biofilm was removed from the membrane, suspended in 10 mL citrate buffer pH 5, 50 mM supplemented with 0.5% v/v Tween 80 and homogenized for 2 × 10 s in homogenizer (DT-50-M-gamma Tube with stirring device, Ultra Turrax, IKA, Germany). The suspension was vortexed at room temperature for 1 h and then centrifuged at 20,000×g, at 4°C. The pellet was resuspended in 10 mL citrate buffer pH 5, 50 mM supplemented with 0.5% v/v Tween 80 and the pooled supernatants were used for the determination of enzymatic activities bound to the biofilm. The pellet was then suspended in EDTA disodium salt (5 g · L^−1^), (Merck, Germany) and centrifuged again at 20,000×g, at 4°C, for 15 min to remove the remaining extracellular polymeric substances (EPS) and then washed with distilled H_2_O and centrifuged at 20,000×g, at 4°C. The washed pellet was used for fungal growth estimation (Supplementary Figure 2).

### Fungal growth estimation

The fungal growth estimation was done by measuring the glucosamine (GlcN) content of the fungal cell wall. The method was based on previous published reports (Ride and Drysdale, 1972; Scotti et al., 2001). The samples were diluted in distilled H_2_O in order to ensure measurements in the linear range of the glucosamine calibration curve and a volume of 0.3 mL was used in the assay. Samples were placed in Pyrex screw capped tubes and 0.3 mL HCl (4M) was used to hydrolyze the fungal biomass. Samples were flushed with nitrogen after HCl addition, and hydrolyzed for 2 h at 121°C. Samples were cooled down and neutralized with 0.4 mL 2M Na_2_CO_3_. After neutralization, 0.5 mL of freshly prepared 2% v/v acetyl acetone in 1.5M Na_2_CO_3_ were added. Samples were heated in a boiling water bath for 20 min and then 1 ml absolute ethanol was added. 0.5 mL of the Ehrlich's reagent [2 g *p*-dimethylaminobenzaldehyde in 30 mL EtOH and 30 mL concentrated HCl (32% w/w)] were used for the colorimetric determination of the glucosamine content. The color formation was measured at 530 nm. A calibration curve using GlcN was constructed to determine the linear range of the measurements. Different calibration curves correlated the cell dry weight (CDW) for each fungus (grown on glucose) with their GlcN content. The influence of the substrates (Avicel and PBWS) on glucosamine measurements was also evaluated within the range of substrate concentrations used in the experiments.

### Enzyme assays

One international unit (IU) of enzyme activity was defined as the amount of enzyme required to liberate 1 μmol of product per min, at assay conditions.EG, CBH and BG activities were expressed as IU · mL^−1^ or as IU · g^−1^ cell dry weight (CDW), or as total IU produced in the reactor. Total units (produced in the reactor) were used to describe enzyme production and also comparisons among biofilm cultivations, because of the localization of the activities in different fractions of the MBM reactor. All enzyme assays were performed in duplicate at pH 5. Filter Paper Activity (FPA) was assayed as described by Wood and Bhat (1988) and expressed as Filter Paper Units (FPU). BG activity was measured using 1 mM *p*np-G (pH 5) as the substrate (0.05 mL sample in a final volume 0.5 mL). The assay was carried out at 50°C for 10 min. The reaction was stopped by the addition of 0.1 mL Na_2_CO_3_ (15% w/v) and the absorbance was measured at 410 nm. EG and CBH assays were carried out as described previously (Xiros et al., 2008) using CMC (2% w/w) and Avicel (1% w/w) as the substrates, respectively, at pH 5 and 50°C.

### Ultrafiltration of enzyme extracts and enzymatic hydrolysis reactions

After harvesting the culture, 0.5% v/v of Tween 80 was added and homogenized for 2 × 10 s (DT-50-M-gamma Tube with stirring device, Ultra Turrax, IKA, Germany). The suspensions were then vortexed at room temperature for 1 h and then centrifuged at 20,000×g, at 4°C. The enzymatic extracts used in the reactions were generated after ultrafiltration of those supernatants using spin filters with a molecular weight cut off of 10 kDalton (Vivaspin®20, Sartorius, Switzerland). The enzymatic profiles of the enzymatic extracts before and after ultrafiltration are shown in Table 1. All enzymatic reactions took place in a thermomixer C (Eppendorf, Hamburg, Germany) at agitation speed 1,400 rpm, in a final volume of 1.5 mL. All reactions were performed in triplicate at pH 5 (phosphate buffer, 50 mM) and T = 30°C, unless otherwise is stated. Dry washed solids from steam pretreated beechwood (PBWS) as well as Avicel were used as substrates (2% w/w).

**Table 1:** Enzymatic profiles of the enzyme extracts produced by biofilm cultivations and used in hydrolysis experiments.

	**CBH**	**EG**	**FPA**	**BG**
**BEFORE ULTRAFILTRATION**				
*T. reesei*	0.26 ± 0.03	14.7 ± 2	2.7 ± 0.4	0.03 ± 0.01
*A. phoenicis − T. reesei*	0.25 ± 0.02	17.2 ± 1.1	2.8 ± 0.3	0.07 ± 0.01
*A. phoenicis*	0.01 ± 0.01	3.0 ± 0.6	0	0.24 ± 0.02
**AFTER ULTRAFILTRATION**				
*T. reesei*	0.70 ± 0.05	81.1 ± 4	6.70 ± 0.6	0.13 ± 0.01
*A. phoenicis − T. reesei*	0.74 ± 0.06	86.0 ± 3.6	6.54 ± 0.5	0.28 ± 0.01
*A. phoenicis*	0.040 ± 0.003	18.2 ± 2.7	0.12 ± 0.06	0.95 ± 0.05

*Cellobiohydrolase (CBH), Endoglucanase (EG) and *β*-Glucosidase (BG) are expressed in IU·mL^−1^ of enzymatic extract, while Filter Paper Activity (FPA) is expressed in FPU·mL^−1^. The values are the averages of three determinations ± the standard deviations*.

### Evaluation of inhibition constants for cellobiose

The effect of cellobiose on cellulose hydrolysis was assessed during hydrolysis reactions of Avicel and PBWS (2 % w/w cellulose in both cases) in citrate buffer pH 5, 50 mM, at 30°C. The reactions were performed in the presence of cellobiose in a range of concentrations from 0 to 30 g · L^−1^. The enzyme loading was 40 FPU g^−1^ of cellulose, in order to assure a significant amount of cellobiose release in a short time and to prevent erroneous results due to the cellobiose added in the reaction. δ-Gluconolactone (3 g · L^−1^) was added to the reaction mixtures to prevent the action of BG; however, the small amounts of glucose formed during the reaction were taken into account (Philippidis et al., 1993). The reaction time was 30 min. The inhibition constants for cellobiose were estimated with nonlinear regression (SigmaPlot v. 12.5) following the equation:

$$\begin{array}{l} r=r_{o}\frac{K_{i}}{K_{i}+C} \end{array}$$

Where, *r*, is the initial reaction rate of cellulose hydrolysis to cellobiose; *r*_*o*_ is the initial reaction rate in the absence of cellobiose; *C*, is the concentration of cellobiose; and *K*_*i*_ is the inhibition constant of cellobiose.

### *In situ* soluble sugars removal

Enzymatic reactions were performed with an enzyme loading of 25 FPU g^−1^ and Avicel initial concentration of 2% w/w, at pH 5 and T = 30 °C. After 24 h of reaction soluble sugars were removed using microtube ultrafilitration membranes (cut off 10 kDalton) under centrifugation at 3,200 rpm at 8°C. After centrifugation, fresh buffer was added and the reaction continued in the initial reaction volume, without adding any fresh enzyme.

### Reducing sugars, glucose, and cellobiose quantification

Reducing sugars were quantified using the DNS method (Miller, 1959). Glucose and cellobiose concentrations were quantified using high performance liquid chromatography (Waters 2695 Separation Module, Waters Corporation, Milford, MA, USA) using an Aminex HPX-87H column (Bio-Rad, Hercules, CA, USA) at 60°C, with 5 mM H_2_SO_4_ as the mobile phase (0.6 mL min^−1^) and a refractive index detector (Waters 410).

### Statistical evaluation of the results

**Figure 1:**
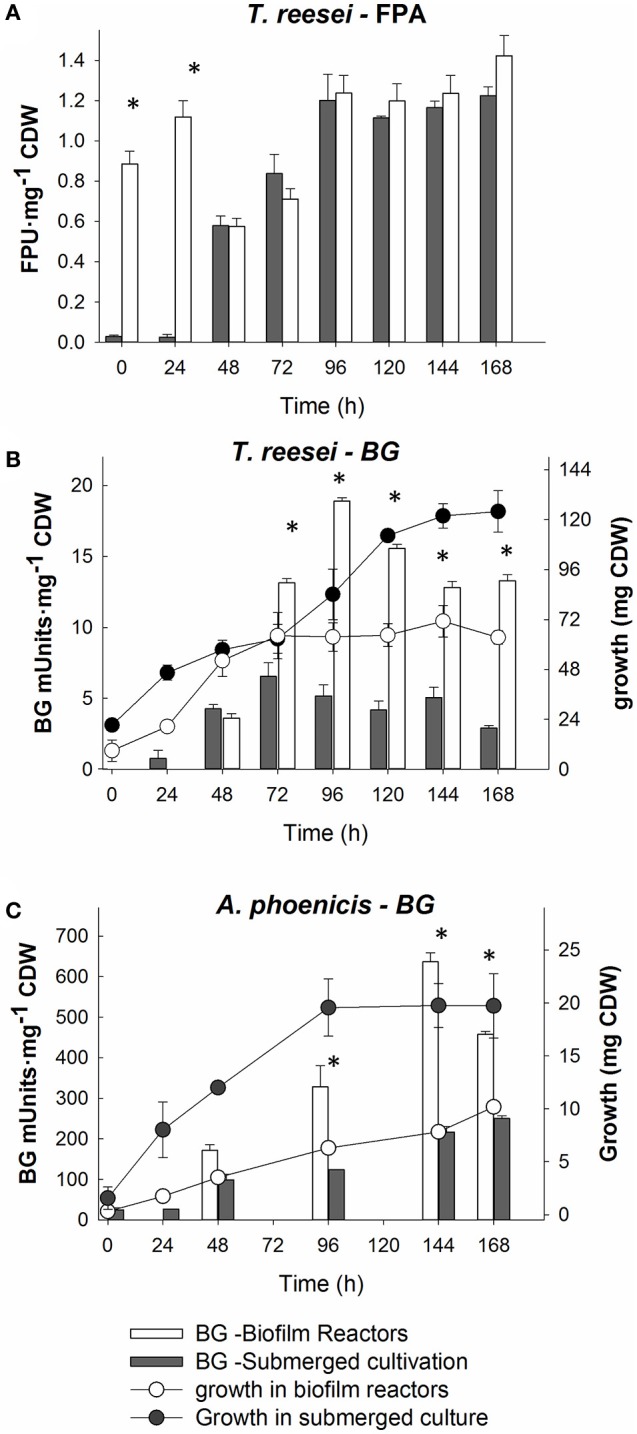
Comparison of cultivation techniques for fungal growth and cellulolytic enzymes production by *T. reesei* and *A. phoenicis*. The comparisons are based on specific enzyme activities (Units per mg of CDW). **(A)** Comparison of Filter paper activity production by *T. reesei* in submerged and biofilm cultivations. **(B)** Comparison of BG activity and growth by *T. reesei* between submerged and biofilm cultivations. **(C)** Comparison of BG activity and growth by *A: phoenicis* between submerged and biofilm cultivations. In all comparisons, equal inoculums and equal culture volumes were used. All cultivations were performed twice, and assays were performed in duplicate. Error bars represent the range between replicates. White color represents biofilm cultures, while gray represents submerged ones. The circles connected with lines show growth, while bars show specific enzyme activities. Only at the time points marked with asterisks, the activities measured were statistically different between submerged and biofilm cultivations.

**Figure 2:**
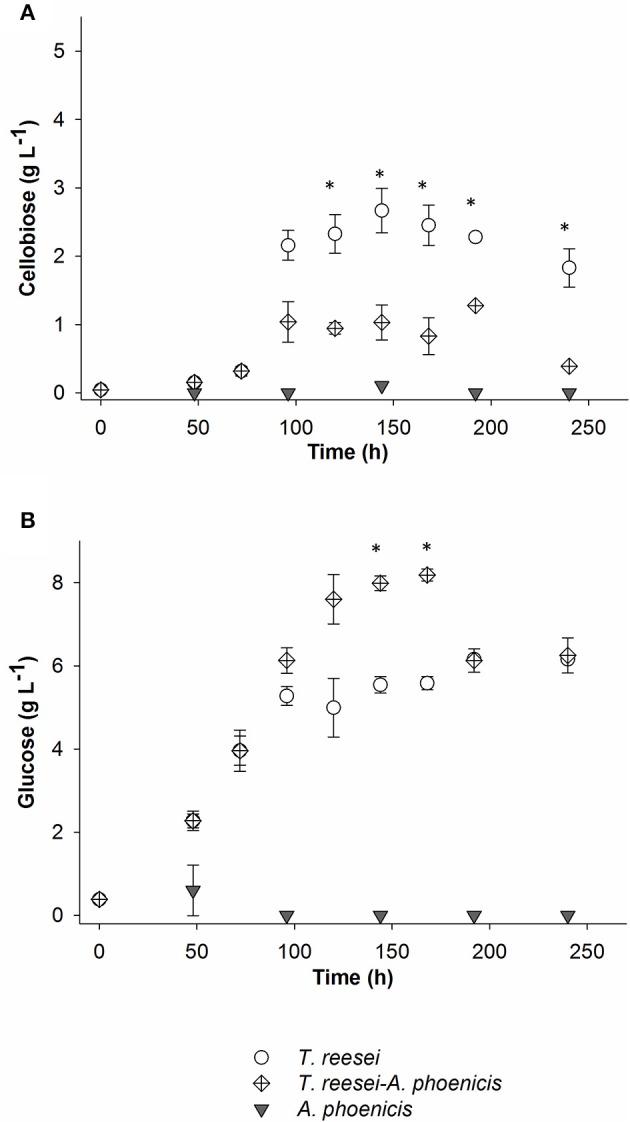
Cellobiose **(A)** and glucose **(B)** concentrations in the liquid fraction of the biofilm cultivations. All cultivations were performed twice. Error bars represent the range between replicates. In the case of the mixed culture, Time = 0 h, corresponds to the inoculation of *T. reesei*. *A. phoenicis* was inoculated 2 days later. The time points marked with asterisks indicate the concentrations that were statistically different between mixed and single (*T. reesei*) cultivations.

**Figure 3:**
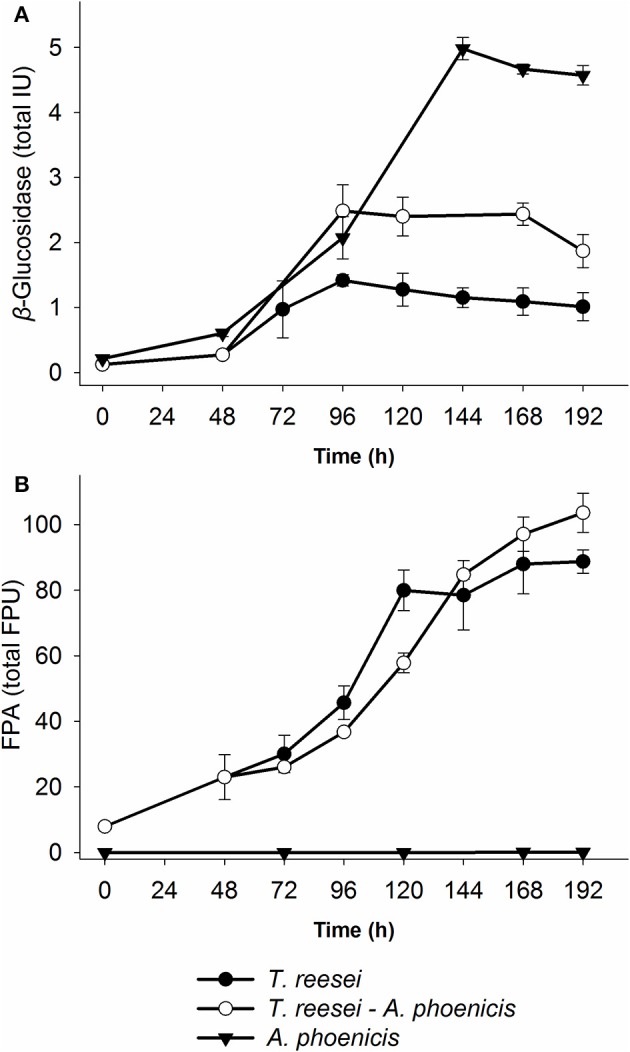
Time course of enzyme production by the single and mixed biofilm cultivations in the biofilm membrane reactors. **(A)** *β*-Glucosidase activity, and **(B)** Filter Paper Activity. In the case of the mixed cultivation, time zero corresponds to the inoculation of *T. reesei*, while *A. phoenicis* was added 48 h later. The activities shown are the sum of all three fractions (Liquid, Biofilm, cellulosic residue) for each time point. Therefore, total units were calculated for the whole MBM reactor. All cultivations were performed twice and enzyme assays were performed in duplicate. Error bars represent the range between replicates. BG activities between the mixed (*A. phoenicis* – *T. reesei*) and the single (*T. reesei*) cultivations measured after 120 h were statistically different. On the contrary, FPA was not found to show statistical differences between the same cultivations. Details on the statistical tests are given in materials and methods section.

**Figure 4:**
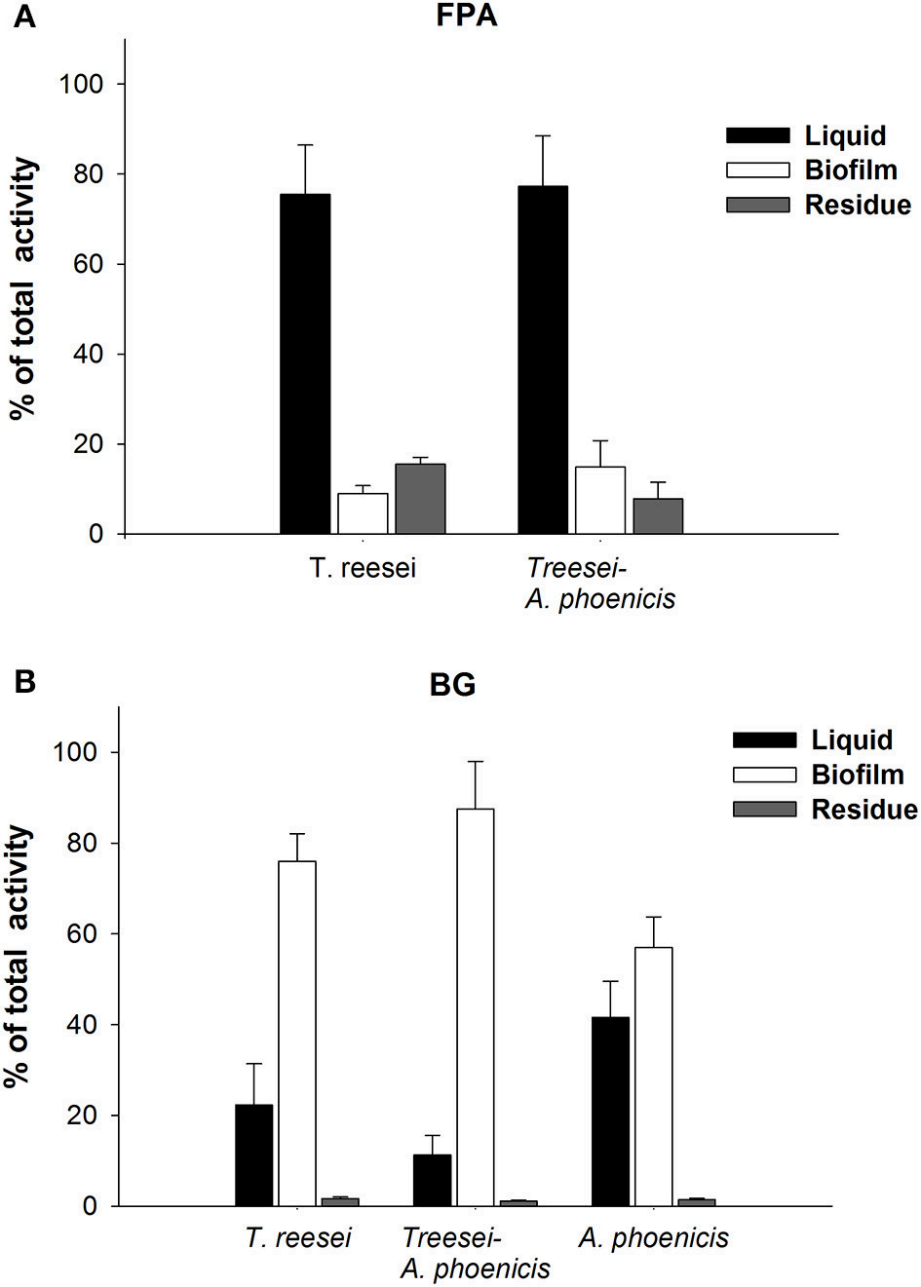
Localization of enzymatic activities in the biofilm membrane reactors during single and mixed cultivations. The activities are given as percentages of the total units produced in the whole reactor. **(A)** FPA, **(B)** BG. All cultivations were performed twice, and assays were performed in duplicate. Error bars represent the range between replicates.

**Figure 5:**
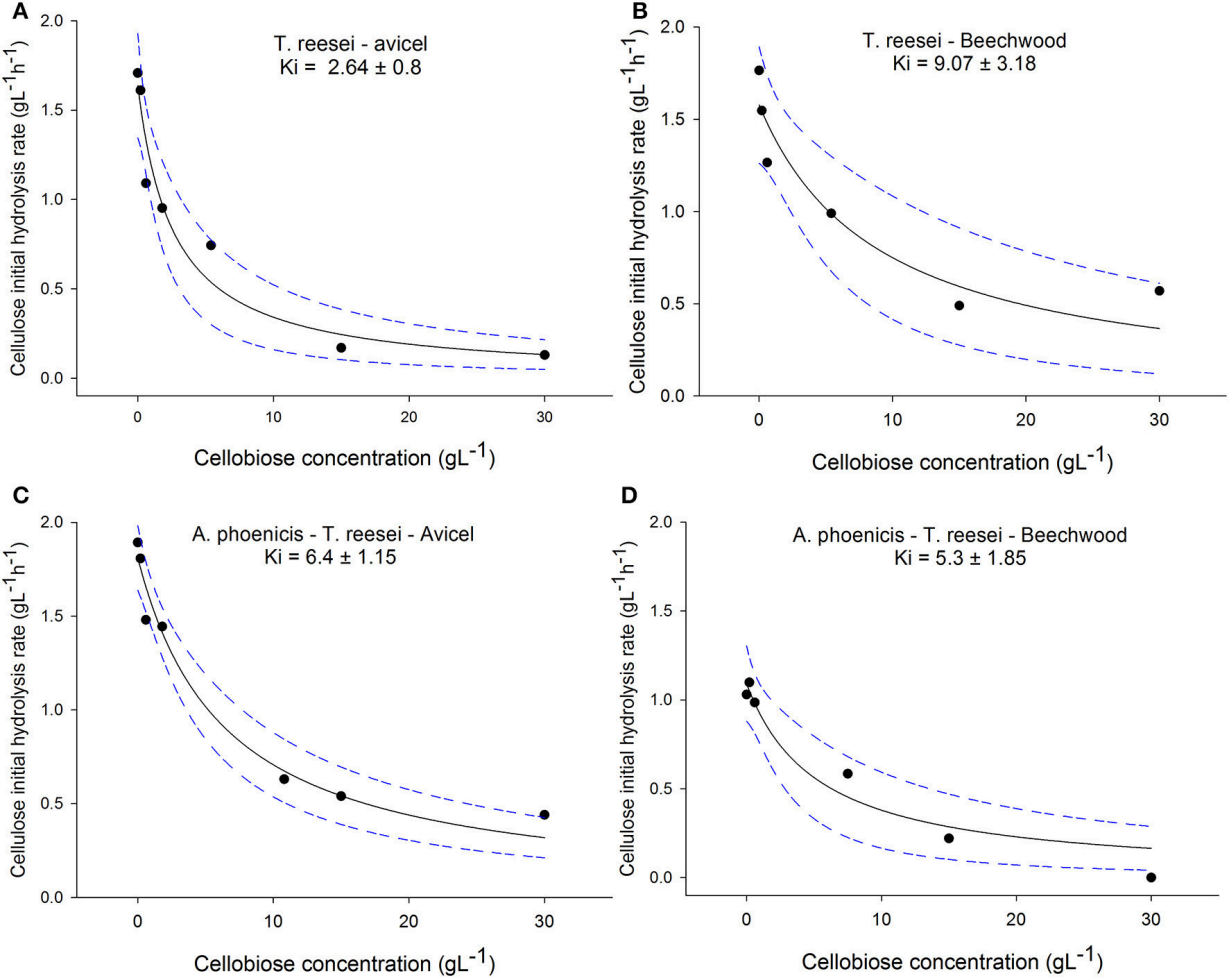
Effect of cellobiose on cellulose initial hydrolysis rates. All reactions were performed in duplicate, with 40 FPU g^−1^ of cellulose, at 30°C and at pH 5. The cellulose loading was 2% w/w in all cases. The *R*^2^ values for the regressions were 0.94, 0.89, for the enzymatic system of *T. reesei* on Avicel **(A)** and PBWS **(B)**, respectively, and for the enzymatic system of the mixed culture (*A. phoenicis* − *T. reesei*) the *R*^2^ values were 0.97 **(C)** and 0.93 **(D)**. The dashed lines show the 95% confidence band. The estimated inhibition constants for the enzymatic system of *T. reesei* were found statistically different between the two substrates for a confidence interval of 80%. No statistical differences between different substrates were found for the enzymatic system of the mixed culture.

**Figure 6:**
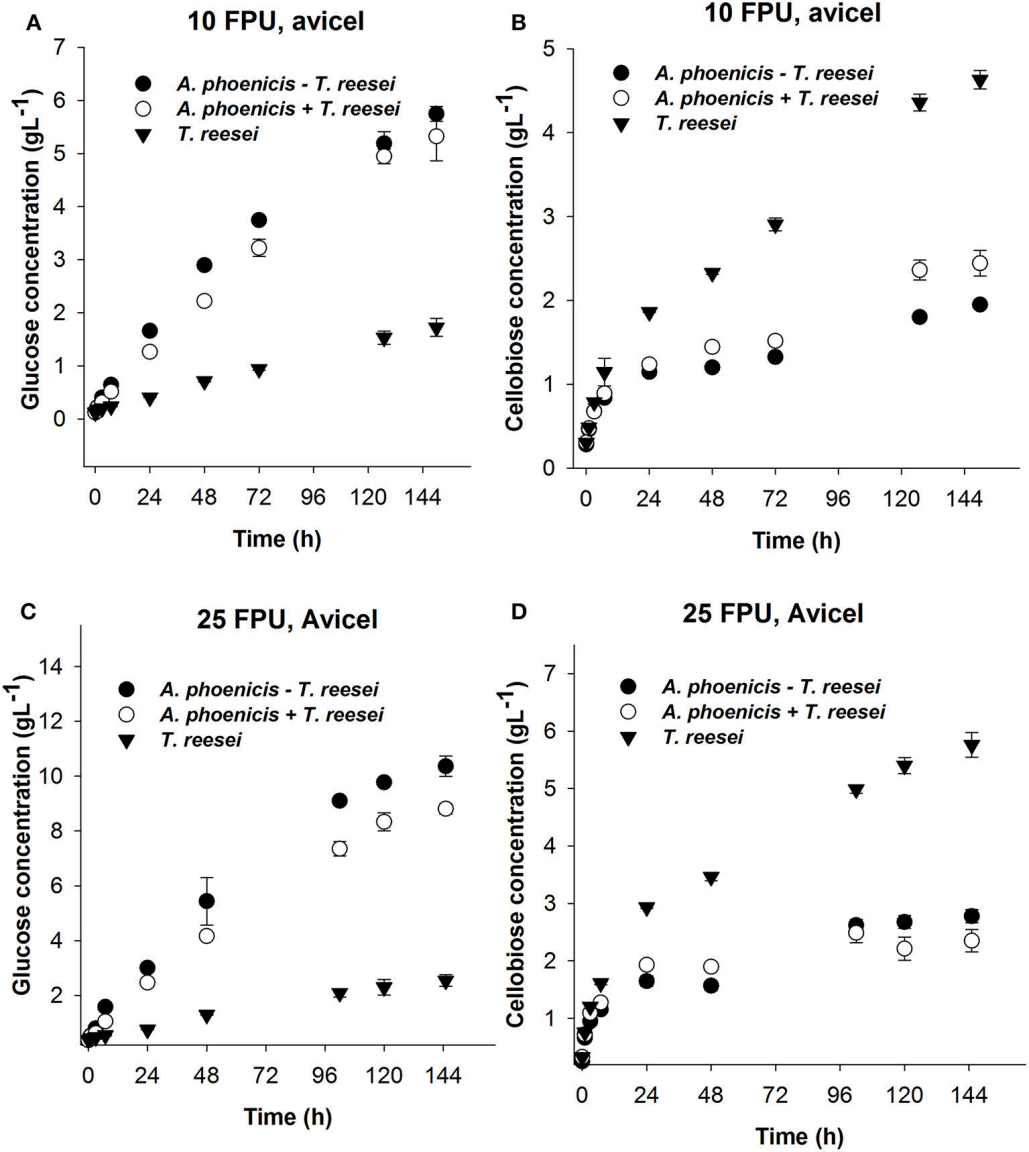
Glucose **(A,C)** and cellobiose **(B,D)** release during enzymatic hydrolysis of Avicel using 10 FPU **(A,B)** and 25 FPU **(C,D)** of enzymatic extract per g of dry substrate. The hydrolytic performance of the extract from the single culture (*T. reesei*) is compared with that from the mixed culture (*A. phoenicis* − *T. reesei*). In the case of *T. reesei* + *A. phoenicis*, the enzymatic extracts of single cultures were mixed to achieve a BG activity equal to that of the mixed culture. All reactions were performed at 30°C, pH 5, in triplicate. Error bars represent standard deviations. Cellobiose and glucose release were compared between the *T. reesei* enzymes and the enzymes of the mixed cultivation. In all cases the differences were found statistically significant. Details on the statistical tests are given in materials and methods section.

**Figure 7:**
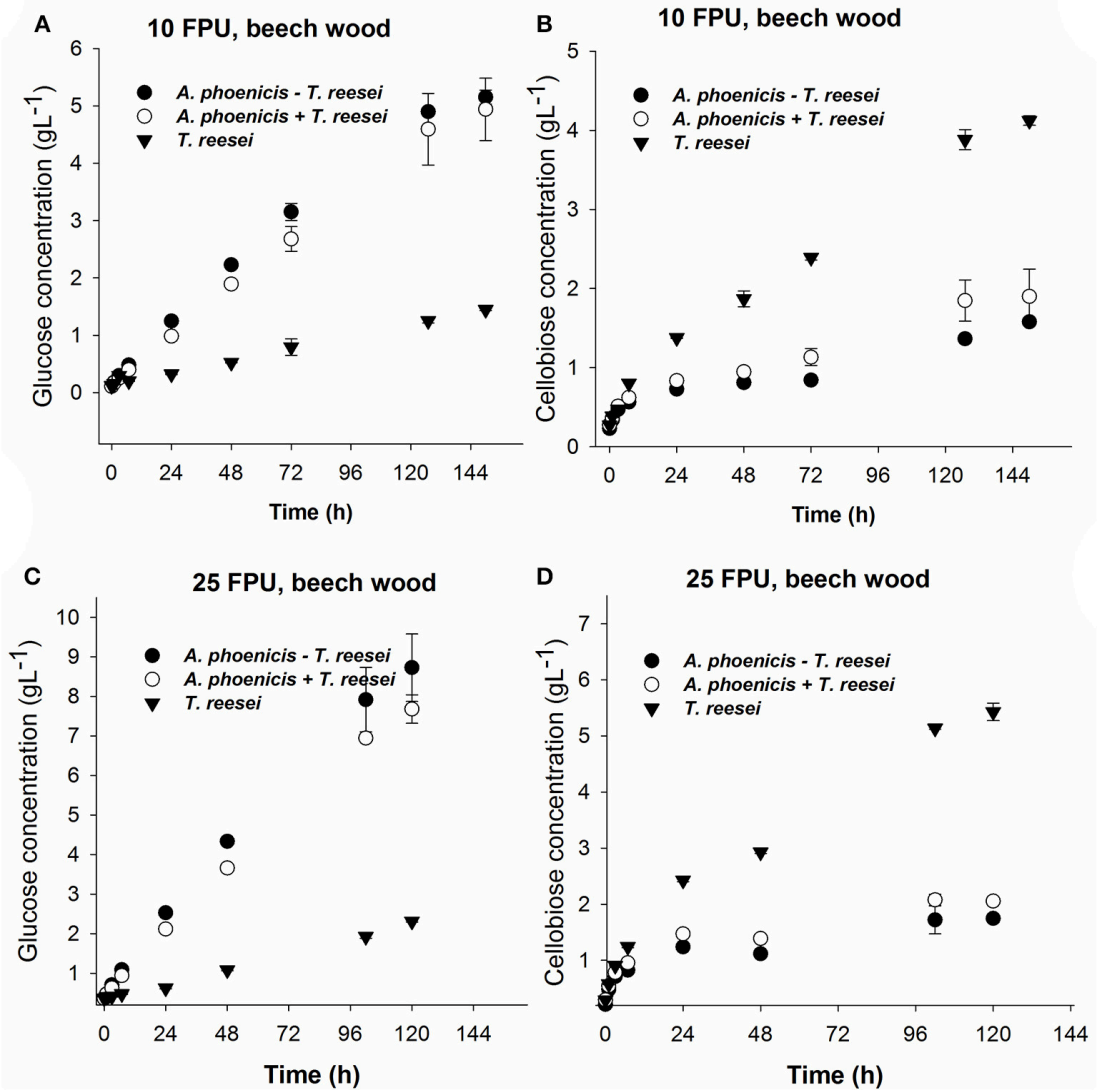
Glucose **(A,C)** and cellobiose **(B,D)** release during enzymatic hydrolysis of PBWS (washed solids from pretreated beechwood) using 10 FPU **(A,B)** and 25 FPU **(C,D)** of enzymatic extract per g of dry substrate. The hydrolytic performance of the extract from the single culture (*T. reesei*) is compared with that from the mixed culture (*A. phoenicis* − *T. reesei*). In the case of *T. reesei* + *A. phoenicis*, the enzymatic extracts of single cultures were mixed to achieve a BG activity equal to that of the mixed culture. All reactions were performed at 30°C, pH 5, in triplicate. Error bars represent standard deviations. Cellobiose and glucose release were compared between the *T. reesei* enzymes and the enzymes of the mixed cultivation. In all cases the differences were found statistically significant. Details on the statistical tests are given in materials and methods section.

**Figure 8:**
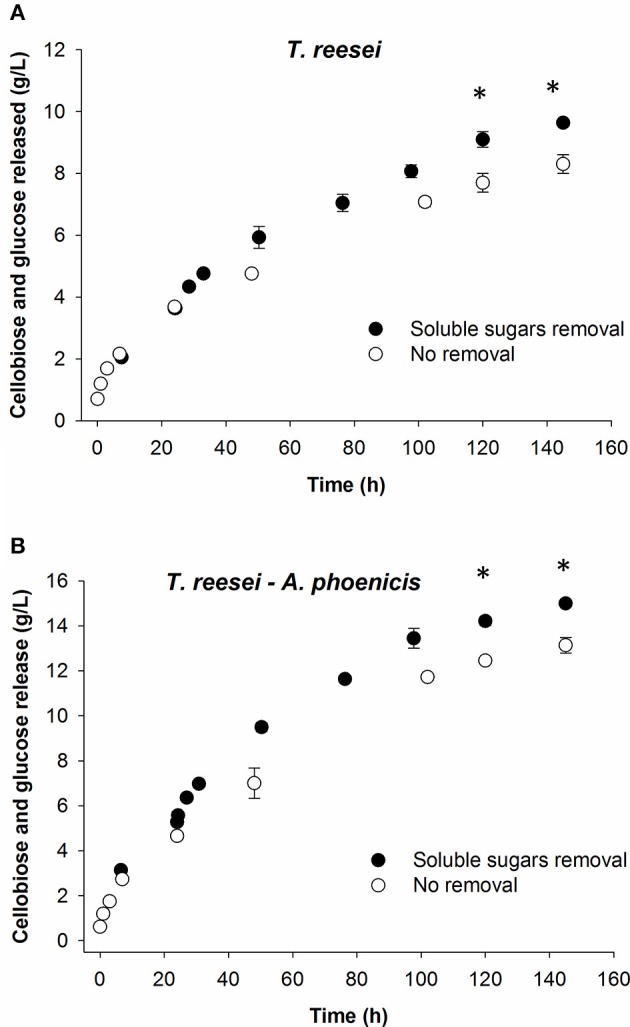
Effect of *in situ* hydrolysate removal on hydrolysis of Avicel by the enzymatic extracts of mixed **(A)** and single cultivations **(B)**. In all cases, the sum of cellobiose and glucose is presented. For the black circles (hydrolysate removal), the calculated released sugars are shown (sugars concentrations measured plus those removed). Reactions were performed at 30°C, pH 5, in duplicate. Error bars represent range between replicates. Sugar release was compared between the removal and no-removal reactions. The statistical significant differences are marked with an asterisk. Details on the statistical tests are given in materials and methods section.

Significant differences between different experimental conditions were evaluated using T-test. A two sided unpaired *t*-test (for independent samples) was applied. Normal distribution was assumed. The T-test was applied for equal variance and 2 degrees of freedom for Figures 1–**3**, **8** and 4 degrees of freedom for **Figures 6, 7** (n1 + n2 − 2). The “t critical” was calculated for DF = 2 or DF = 4 and probability = 0.05. The null hypothesis was that the groups compared were not different. The null hypothesis was rejected when the absolute *t* stat value was higher than the *t* critical value. In the figures the error bars represent the range of the two independent values in case of two replicates; while the standard deviation is shown in the cases where triplicate measurements were performed. For cellobiose inhibition (**Figure 5**), the significant difference of the constants calculated by the models was evaluated for 80% confidence intervals.

## Results

### Cellulolytic enzymes production by fungal biofilms

#### Comparisons between submerged and biofilm cultivations regarding growth and enzyme production

*T. reesei* RUT C-30, a filamentous fungus widely used for cellulolytic enzymes production, was grown at submerged and biofilm cultivations, using Avicel (2% w/w) as the carbon source. The fungal growth and also the cellulolytic enzymes production were compared between the two cultivation methods. The comparison was based on fungal growth and extracellular FPA and BG productions. Total FPU (for FPA) and total IU (for BG) for equal culture volumes were calculated. At submerged conditions, higher enzyme activities per culture volume were measured (data not shown). However, as shown in Figures 1A,B, where the enzyme activities were normalized based on fungal CDW, the higher activities at submerged conditions were mainly due to the significantly higher fungal growth at these conditions.

The comparison between the two cultivation techniques indicates that when *T. reesei* grew in a biofilm, the cells showed an enhanced metabolic activity. The production of both FPA and BG practically stopped after 96 or 120 h of cultivation in all cases. In the biofilm membrane reactors growth already stopped after 72 h. However, BG activity remained at very low levels (below 45 mU mL^−1^) in both cultivations. The low BG productivity was also reflected on the cellobiose levels in the liquid medium, measured during the cultivation in the membrane reactor. As shown in Figure 2A, cellobiose accumulated in the MBM reactors after 96 h of cultivation.

During the experiments with *A. phoenicis* as the sole microorganism, the formation of biofilm by *A. phoenicis* in the MBM system was confirmed only when glucose was used as the carbon source. In these experiments glucose and Avicel were tested as carbon sources. In the case of Avicel, the biofilm formation was very slow and not so clear. In fact, a very thin and not well formed biofilm was observed only after 5 or 6 days of cultivation. These observations reflected and confirmed the weak growth of *A. phoenicis* on Avicel (Figure 1C). *A. phoenicis* could not efficiently hydrolyze Avicel and grow on it, as indicated by the overall cellulase activity (expressed as FPA) measurements, which were close to the detection limits of the method used (Table 1 and Figure 3). As also shown in Table 1, there was a lack of CBH activity in *A. phoenicis* enzyme extract which explained the inability of the fungus to grow well on Avicel. Fungal growth in the membrane reactors was half of that in submerged cultivation, but as in the case of *T. reesei*, cells were metabolically very active as shown by the enhanced specific BG production compared to the submerged cultivation. As shown in Figure 1C, a three-fold difference in BG activity normalized on fungal growth (CDW) was observed after 4 days of *A. phoenicis* cultivation. BG activity after 6 days of cultivation was higher in the case of membrane reactors even when activities per culture volume were measured.

#### Localization of enzymatic activities in biofilm membrane reactors

Culture supernatants have been used for enzymatic activities estimation during experiments at submerged conditions in numerous studies. In SSCs of fungi, an extraction step is necessary to obtain and measure the produced (extracellular) activities (Xiros et al., 2008). Fungal biofilm cultivations have been considered as an intermediate between solid and submerged cultures: Fungi grow on a solid surface but completely covered by the liquid medium. In the present study, the activities measured in the liquid supernatants from the cultivations represented only a part of the cellulolytic enzyme production. The measurements of enzymatic activities in all three fractions (solid residue, biofilm, liquid medium) of the cultures revealed the true amounts of cellulolytic activities produced (Figure 4). The distribution of activities among these fractions did not remain constant, but depended on the activity measured as well as on the cultivation time. In Supplementary Figure 1 the localization of Filter Paper and *β*-Glucosidase activities over time is shown for *T. reesei* cultivations. As shown, the distribution was different for BG and FPA. BG activity increased over time in the biofilm fraction while it decreased in the liquid. Taking into account all three fractions, it can be observed from Figure 1 that BG production stopped 1 day after fungal growth reached its maximum value. BG activity increased until 96 h and changed only slightly thereafter. On the contrary, the general cellulolytic activity (FPA) increased in the liquid, as the cultivation time passed, possibly also due to cell lysis during the late stage of cultivation. However, FPA does represent a mixture of cellulolytic activities, and thus conclusions regarding its relation with growth are difficult.

### Enhancement of *β*-glucosidase production by multispecies biofilm formation in membrane reactors

#### Setup of *T. reesei* and *A. phoenicis* co-cultivation

In preliminary experiments, three different inoculation procedures were tested: (a) Subsequent inoculation with *T. reesei* as the first microorganism, (b) subsequent inoculation with *A. phoenicis* as the first microorganism, and (c) simultaneous inoculation of the two fungi. The three setups were evaluated with regard to the cellulolytic activities produced. When *T. reesei* was the first microorganism inoculated and *A. phoenicis* was added 2 days afterwards, the best results in terms of enzyme production were obtained. This was expected as in this case, *A. phoenicis*, due to its enhanced *β*-Glucosidase production, could grow on the cellobiose which had been already released by *T. reesei* enzymes in the medium. Microscopic observations of the biofilms were performed for all different setups. However, both *T. reesei* and *A. phoenicis* in the biofilm could microscopically be observed when *T. reesei* was the first microorganism and *A. phoenicis* was added afterwards.

In single microorganism experiments, *T. reesei* formed a distinct biofilm after 48 h. On the contrary, *A. phoenicis* barely grew on Avicel due to the inability of this fungus to grow on cellulose as it lacks a complete cellulolytic enzymatic mixture. When both fungi were simultaneously inoculated in the MBM system, *A. phoenicis* hyphae could not be observed in the biofilm during microscopic observation and no differences were observed in BG production. The inoculation of *A. phoenicis* 2 days after *T. reesei* was found to be a much better procedure for the co-cultivation. However, a more detailed investigation would probably indicate more precisely the optimum inoculation time of *A. phoenicis* and would further enhance the BG production.

#### Enzyme production in multispecies biofilm membrane reactors

In Figure 3, the enzymatic activities produced by the multispecies biofilm cultivations are compared with the single biofilm ones. As shown, there was a significant enhancement of BG activity produced by the mixed culture, compared to *T. reesei* alone: An increase by a factor of 2.5 was achieved after 2 days of common cultivation (corresponding to 4 days from *T. reesei* inoculation). The maximum BG activity was 2.5 IU for the whole reactor (Figure 3), corresponding to 0.084 IU·mL^−1^. However, BG activity did not reach the levels of the *A. phoenicis* single culture (5 IU). The increased BG activity was also reflected to the cellobiose concentrations during the cultivation. Cellobiose concentration decreased by 50 to 70% and remained at low levels (about 1g·L^−1^) throughout the experiment (Figure 2). FPA activity was only slightly affected by the addition of *A. phoenicis* and reached about 85% of its maximum value after 4 days of co-cultivation (Figure 3).

The microscopic observation of the mixed biofilms showed that there were distinct regions where the presence of one fungus was more intense and regions where the distribution of the two fungi was more balanced. Although a quantitative analysis of the microscopic images was not possible, it was clear that the distribution of the two fungi in the biofilm was changing overtime. The activities measured in the biofilm fraction (Figure 4 and Supplementary Figure 1) also showed differences related not only to the microorganisms involved but also related to the cultivation time. FPA was mainly found in the liquid fraction, while the amount adsorbed on the residual solids (Avicel) was changing overtime, and on the 7th day of the cultivation it only corresponded to 16 and 8% of the total FPA measured in all fractions (Figure 4), for *T. reesei* and the mixed culture (*A. phoenicis* – *T. reesei*), respectively. As also shown in the same figure, after 168 h of growth, BG activity was found mainly in the biofilm, except for the case of *A. phoenicis* single culture, where almost half of the activity was measured in the liquid fraction. However, this was expected, as in this case the biofilm was not well formed.

### Hydrolytic efficiency of enzymes secreted by single- and multi-species biofilms

#### Inhibition of cellulases by cellobiose

The effect of cellobiose on cellulose hydrolysis was studied for both cellulolytic systems (produced by *T. reesei* and by the mixed culture *A. phoenicis* – *T. reesei*) and for two types of cellulosic substrates (Avicel and PBWS). As can been seen in Figure 5, the regressions successfully described the effect of cellobiose on cellulases, with *R*^2^ for all cases ranging between 0.89 and 0.97. In the case of *T. reesei* enzymes, the estimated values for the inhibition constants varied between the two substrates (2.64 g · L^−1^ for Avicel and 9 g · L^−1^ for PBWS). The difference between the two constants was statistically significant for confidence intervals 80%. No significant difference between the substrates was observed in the case of the *A. phoenicis* – *T. reesei* enzymes.

#### Hydrolysis of different cellulosic substrates using different enzyme dosages

Hydrolysis reactions were designed and performed to evaluate the hydrolytic efficiency of the enzymatic systems produced by single and mixed cultures. The reactions were performed at the cultivation conditions (30°C, pH 5) in order to be able to derive lessons for a consolidated bioprocess based on a microbial consortium where fungi grow and secrete enzymes to release sugars fed to the fermenting microorganism in the anaerobic part of the reactor (Brethauer and Studer, 2014). Apart from Avicel, PBWS were also used as substrate. Analysis of PBWS for cellulose and acid insoluble lignin contents, gave mass fractions of 60.5% w/w (±5.9%) and 36.7 % w/w (±1.4%), respectively, on a dry weight basis. All hydrolysis reactions were performed with a substrate concentration of 2% w/w.

During Avicel hydrolysis, 10 FPU·g^−1^ of *T. reesei* enzymes hydrolyzed 17% of the substrate in 72 h, while 6.4 g·L^−1^ of glucose and cellobiose cumulatively were released after 150 h of reaction corresponding to a hydrolysis yield of 29%. However, cellobiose concentration reached 4.7 g·L^−1^, which was almost three times higher than the glucose released (1.7 g·L^−1^) at the same reaction time (Figure 6). The picture of released sugars was similar during hydrolysis of PBWS using the same amount of *T. reesei* enzymes, where 41% of total cellulose was hydrolyzed after 150 h, releasing 5.5 g·L^−1^ of glucose and cellobiose cumulatively, while glucose concentration reached 1.6 g·L^−1^ corresponding to less than half of the cellobiose concentration (3.9 g·L^−1^) (Figure 7).

The enzymatic system produced by the mixed biofilm of *A. phoenicis* and *T. reesei* was more efficient in hydrolyzing both Avicel and PBWS. Using 10 FPU of that enzyme extract, 7.7 g·L^−1^and 6.7 g·L^−1^ of glucose and cellobiose were cumulatively measured after 150 h hydrolysis of Avicel and PBWS, respectively (Figures 6, 7). In both cases and due to the enhanced BG activity, the released glucose was dramatically increased compared to the glucose release by only *T. reesei* enzymes: 5.7 g·L^−1^ and 5.2 g·L^−1^ of glucose was released from Avicel and PBWS, respectively, corresponding to 80–90% of total sugars released.

As expected, the use of higher enzyme dosage increased the initial hydrolysis rates (Table 2), and also the final hydrolysis yields. When 25 FPU/g solids were used in the reactions, the initial rates were at least doubled in all cases. The initial reaction rates also revealed the difference in digestibility between Avicel and PBWS. In all cases initial reaction rates of PBWS hydrolysis were significantly lower than those of Avicel hydrolysis. The released glucose from Avicel reached 10.4 g·L^−1^ and 2.5 g·L^−1^ for the enzymatic extracts from the mixed (*A. phoenicis* − *T. reesei*) biofilm and the single one (*T. reesei*), respectively. Comparing with the hydrolysis reactions with 10 FPU·g^−1^, the increase in glucose release was much higher in the case of the extract from the mixed biofilm, confirming that in the case of *T. reesei* extract, the crucial weakness is the lack of BG activity and not the enzyme dosage. The sum of glucose and cellobiose after 120 h of hydrolysis was 13.1 g·L^−1^ for the enzymes from mixed culture, and 8.3 g·L^−1^ for those from the single culture. In the case of PBWS, the sum of these two sugars reached 10.5 g·L^−1^ corresponding to 79% of cellulose content (Figures 6, 7).

**Table 2:** Initial reaction rates during hydrolysis of Avicel and PBWS.

**Enzymatic system**	Initial reaction rates (g^*^L^−1^^*^h^−1^)			
	10 FPU^*^g^−1^		25 FPU^*^g^−1^	
	**Avicel**	**PBWS**	**Avicel**	**PBWS**
*T. reesei*	0.24	0.14	0.49	0.32
*A. phoenicis − T. reesei*	0.27	0.18	0.58	0.38
*A. phoenicis* + *T. reesei*^a^	0.26	0.15	0.54	0.36

*All reactions were performed using a substrate concentration of 2% w/w. The initial rates were based on the sum of cellobiose and glucose concentrations, which were measured 1 h after the enzymes addition*.

a *An amount of enzyme extract from single A. phoenicis cultures was added to the enzymatic extract of T. reesei (denoted as A. phoenicis + T. reesei). After the addition, the BG activity of the mixture was equal to the one in the extract from the A. phoenicis − T. reesei co-culture*.

#### Effect of *in situ* sugar removal during enzymatic hydrolysis

In a consolidated bioprocess using the MBM reactor, the sugars released due to the action of fungal enzymes, are consumed mainly by the fermenting microorganism, resulting in the formation of the final products (Brethauer and Studer, 2014). Thus, like in simultaneous saccharification and fermentation, the sugars quickly disappear from the cultivation medium. To evaluate the hydrolytic efficiency of the different enzymatic systems at such conditions, hydrolysis reactions with *in situ* sugars removal were conducted. As shown in Table 3, a significant enhancement in reaction rates was observed right after the sugars removal as a result of decreased end product inhibitory effects on hydrolytic enzymes. The reactions rates decreased rapidly during the following 24–48 h of reactions. However, compared to the reactions where no *in situ* sugars removal was performed, the final hydrolysis yields increased 9 and 16% for the reactions with enzymes from mixed biofilm (*A. phoenicis* − *T. reesei*) and from the single one (*T. reesei*), respectively (Figure 8).

**Table 3:** *In situ* sugars removal during hydrolysis of Avicel by the enzymatic systems produced by single (*T. reesei)* and mixed cultures (*A. phoenicis* − *T. reesei*).

**Enzymatic system**	**Sugar**	**Before removal (gL^−1^)**	**After removal (gL^−1^)**	**Sugars removed (%)**	**Reaction rate increase (%)**
*T. reesei*	Cellobiose	2.72 ± 0.11	1.76 ± 0.07	35	88
	Glucose	0.93 ± 0.08	0.53 ± 0.06	42	
*A. phoenicis − T. reesei*	Cellobiose	2.05 ± 0.04	1.24 ± 0.02	39	91
	Glucose	3.13 ± 0.16	1.50 ± 0.16	52	

## Discussion

### Evaluation of *T. reesei* and *A. phoenicis* biofilms for cellulolytic enzymes production

As shown in Figure 1, both fungi grew better in submerged cultures than in biofilm membrane reactors. Although the enzymatic activities produced per volume of culture were significantly higher in the case of the submerged cultures, the FP and BG specific activities (activities normalized per CDW) were higher in the case of the MBM system (biofilm growth). Due to the biofilm membrane reactor design, growth is closely related to the available membrane area. Increase in the area (and more specifically increase in the ratio of membrane area to reactor volume) would result in increased fungal growth in terms of CDW and therefore it would further increase enzyme production. However, it is difficult to predict how it would affect specific enzymatic activities as activity is not linearly correlated with concentration. These findings are in accordance with previous studies showing that the cultivation technique influences the fungal morphology. The morphology in turn, affects fungal productivities and metabolism (Wösten et al., 1991; Quintanilla et al., 2015). Villena and Gutierez-Correa compared the specific enzyme activities produced by another *Aspergillus* species (*A. niger*) in submerged and biofilm cultivations, and showed that biofilm cultures achieved much higher specific activities (Villena and Gutierrez-Correa, 2007). In that case, as also in the present study, the different morphology of fungi at different cultivation conditions reflected differences in metabolism, which in turn, resulted in enhanced specific enzymatic productivities during biofilm growth. Thus, growth (rate and yield) and cultivation technique are two crucial parameters for the optimization of enzyme production by fungi.

It is well known from literature that the production of cellulases by fungi is associated with their growth. In batch cultivation, most cellulolytic activities are produced during the exponential or the early stationary phase (Xiros et al., 2008; Xiros and Christakopoulos, 2009). This is reasonable, since the fungus needs to degrade the substrate to gain the necessary carbon, and to do so, it needs to produce lignocellulolytic enzymes. This was partially observed during this study, as shown in Figures 1, 3, for both fungi and both enzyme activities measured over time: Only a slight increase in measured activities was observed after 100 (BG) or 120 h (FPA) of cultivation, while fungal growth reached highest levels 24 h or 48 h before that. Besides, the lack of major cellulolytic activities in the secretome of *A. phoenicis* (Table 1) resulted in very limited growth of this fungus on Avicel (Figure 1). This observation is in accordance with previous reports showing the inability of this fungus to produce a complete cellulolytic enzymatic mixture, capable of hydrolyzing cellulosic substrates (Wen et al., 2005). Therefore, *A. phoenicis* has rarely been studied as the sole microorganism in cultures using cellulose. Instead, lignocellulosic substrates also containing small amounts of free sugars like sugar beet pulp are more suitable for its growth (Deschamps and Huet, 1984).

### Enhancement of BG activity

Many *Aspergillus* species, including *A. phoenicis* have been used in co-cultures in order to enhance the production of BG activity (Castillo et al., 1994; Wen et al., 2005; Brijwani et al., 2010). However, a co-cultivation of *A. phoenicis* with *T. reesei* in biofilms had never been tried before. As also previously implied by Brethauer and Studer (2014) the low BG activity produced by *T. reesei* in the MBM system would lower the efficiency of the consolidated bioconversion system for any cellulose containing plant biomass. Therefore, a multispecies approach was applied during this study in order to enhance the BG productivity in the system. *A. phoenicis* a *β*-Glucosidase overproducer (Woodward and Wiseman, 1982) was selected to form a multispecies biofilm with *T. reesei*. The two fungi have similar optimal values for growth *pH* and *T* and also both microorganisms have been successfully co-cultivated before, in Mandel's medium (Woodward and Wiseman, 1982; Wen et al., 2005).

The progressive accumulation of glucose in the liquid medium was a strong indication that fungal anabolism slowed down and thus glucose was not assimilated by the fungi (Figure 2B). The fact that this happened not only in the case of *T. reesei*, but also in the case of the mixed cultivation where cellobiose levels were low, may imply that the cellulolytic rates were higher than the sugar uptake rates for this strain at these conditions. The enhancement of BG activity during the co-cultivation did not significantly affect FPA, which is in accordance with previous studies (Wen et al., 2005).

### Hydrolytic efficiency of the enzymatic systems

The enhancement of BG in enzymatic extract from the mixed culture affected sugars release from both substrates used, during the enzymatic reactions performed. The effects on glucose release were spectacular and these were reflected to a smaller extent on total sugars release. The effects of BG enhancement on the initial reaction rates were more visible when 25 FPU g^−1^ were applied as enzyme loading. In this case, the enzymatic extract of the mixed culture achieved 16% higher reaction rates than the one from *T. reesei* for both Avicel and PBWS. As observed, the reaction rates decreased rapidly after the first 10–20 h, but only slightly thereafter. The initial decrease in the rates was more evident in the case of PBWS. This implies structural unevenness in this substrate compared with the structurally more homogeneous Avicel (Xiros et al., 2011). The reactions took place at 30°C in order to simulate the conditions during the CBP process in the MBM reactor. At this temperature, the enzymes remained active for quite a long time, but the relatively low reaction rates would be a bottleneck for a commercial process. It is clear, that the use of thermotolerant fungi and fermenting microorganisms would boost the productivity of the system, allowing hydrolysis to take place in higher temperature. A higher reaction temperature would increase the initial rates and shorten significantly the reaction time, but could also result in a faster deactivation of the enzymes.

The initial reaction rates when 25 FPU·g^−1^ were used as the enzyme loading, are roughly double than the rates with 10 FPU·g^−1^ (Table 2) but not proportional to the enzyme dosage increase. This not proportional relationship (A Langmuir-like dependence) reflects the functional adsorption of enzymes on the cellulose. Beyond a certain enzyme loading, no further significant increase in the initial rates can be observed due to the saturation of the substrate with enzymes (Xiros et al., 2011). As shown in Table 2, initial reaction rates were much higher for Avicel hydrolysis than for PBWS. This was probably due to the adsorption (not functional binding) of enzymes on lignin in the case of PBWS leading to decreased amounts of active enzyme molecules. The differences in initial reaction rates between PBWS and Avicel hydrolysis reactions would decrease as enzyme dosage would increase, because lignin (in PBWS) would become more and more saturated with enzymes. The enzyme loading of 25 FPU·g^−1^ is somewhat less than half of the FPA in the biofilm reactors. Of course, a comparison between the two processes (hydrolysis and cultivation) is not possible, due to the different localization of the enzymes in the two systems and also due to the existence of active cells in the second case which metabolize the released sugars.

The substrate used in the cultivations is also of importance, since cellobiose inhibition on cellulases depends not only on the present enzymatic activities but also on the kind of cellulose that is hydrolyzed. Gruno et al. (2004) reported that cellobiose had much stronger inhibitory effects on enzymes that act on crystalline regions (CBHs) than on enzymes that act on amorphous cellulose (EGs). This is also reflected to the differences in the values of cellobiose inhibition constants on cellulases that had been reported for different substrates, and vary from 0.5 g·L^−1^ to 5.5 g·L^−1^ (Philippidis et al., 1993; Gruno et al., 2004). However, the *K*_*i*_ values in the literature are strongly depended on the reaction conditions and therefore they can hardly be compared. During the present study the significance of the substrate for cellobiose inhibition was confirmed in the case of *T. reesei* enzymes: When Avicel was used as the substrate, the *K*_*i*_ for cellobiose was almost four times lower than during PBWS hydrolysis (Statistically confirmed in 80% confidence intervals). Indeed, Avicel is a very crystalline form of cellulose, and its hydrolysis is very much depended on the CBH activity of the cellulolytic system. PBWS on the other hand was generated during steam pretreatment from beechwood, and contains amorphous regions which are hydrolyzed mainly by EGs. In the case of Avicel hydrolysis by *T. reesei* cellulases, the *K*_*i*_ value (2.64 g·L^−1^) showed that the cellobiose concentrations measured after 96 h of cultivation in MBM reactors (using Avicel as the carbon source), decreased the cellulolytic rate about 50%. On the other hand, BG production by the mixed biofilm (*A. phoenicis* − *T. reesei*) limited cellobiose accumulation up to 1.3 g·L^−1^. At these levels cellobiose could have decreased hydrolysis rates only by 20% (*K*_*i*_ = 6.4 g·L^−1^).

*In situ* sugars removal almost doubled the reaction rates. End-product inhibition of lignocellulolytic enzymes has been regarded as a major bottleneck of many bioconversion processes. However, during the present study, only slightly over one third of the cellobiose was removed. The cellobiose concentration in the reaction mixtures increased again quickly and contributed to a rapid decrease in reaction rates. This could be one reason for the moderate effect that the hydrolysate removal had on the final saccharification yields in both cases. Moreover, it should be kept in mind that inhibition by cellobiose does not give the whole picture of the inhibition, as various hydrolysis products have inhibitory effects on the hydrolytic enzymes including oligosacharides, which were not quantified before and after the removal (Kim et al., 2011; Xiros et al., 2011).

## Conclusion

This study showed the efficiency of fungal biofilms as cellulolytic enzyme producers using two filamentous fungi *T. reesei* and *A. phoenicis*. The accumulation of cellobiose when *T. reesei* was the sole enzyme producer showed that there was a lack of BG activity in the system. A multispecies approach was applied using *A. phoenicis* as the BG producer microorganism. Although *A. phoenicis* could not grow on Avicel as the sole microorganism, due to the lack of CBH activity in its secretome, it was possible to form a multi-species biofilm with *T. reesei* in the MBM reactors, proving the versatility of the reactor design to host multi-species fungal biofilms. It was shown that fungal biofilms are effective systems for cellulolytic enzymes production compared to other cultivation techniques. The two fungi co-formed a biofilm, which produced a balanced cellulolytic system containing all main activities (CBHs, EGs, BGs) for cellulose hydrolysis. From the results of the enzymatic hydrolysis reactions on Avicel and PBWS by *T. reesei* enzymes, it was shown that the inhibition of cellulases by cellobiose was substrate depended. It was also concluded that the enhancement of BG activity (in the secretome of the multispecies biofilm) compared to *T. reesei* enzymatic system, not only was important for the glucose yields but also could positively affect the initial hydrolysis rates.

## Author contributions

CX conceived the study, conducted the experiments, and drafted the manuscript. CX and MS performed the experimental design and the analysis of results. MS and CX critically revised the manuscript and both authors approved the final version of the article.

### Conflict of interest statement

The authors declare that the research was conducted in the absence of any commercial or financial relationships that could be construed as a potential conflict of interest.

## Abbreviations

CDW: Cell Dry Weight
BG: *β*-Glucosidase
DM: Dry matter
EG: Endoglucanase
CBH: Cellobiohydrolase
FPA: Filter Paper Activity
PBWS: Washed Solids from Pretreated Beechwood
MBM (reactors): Multispecies Biofilm Membrane reactors
SSC: Solid State Cultivation
CBP: Consolidated bioprocess
HCBP: Highly Consolidated Bioprocess
NREL: National Renewable Energy Laboratory
DM: Dry matter
PDA: Photodiode array.
