# Supplementary figures and images for: A Multispecies Fungal Biofilm Approach to Enhance the Celluloyltic Efficiency of Membrane Reactors for Consolidated Bioprocessing of Plant Biomass
Source: Front Microbiol. 2017 Oct 10;8:1930. doi: 10.3389/fmicb.2017.01930 (PMC5641325; doi:10.3389/fmicb.2017.01930)

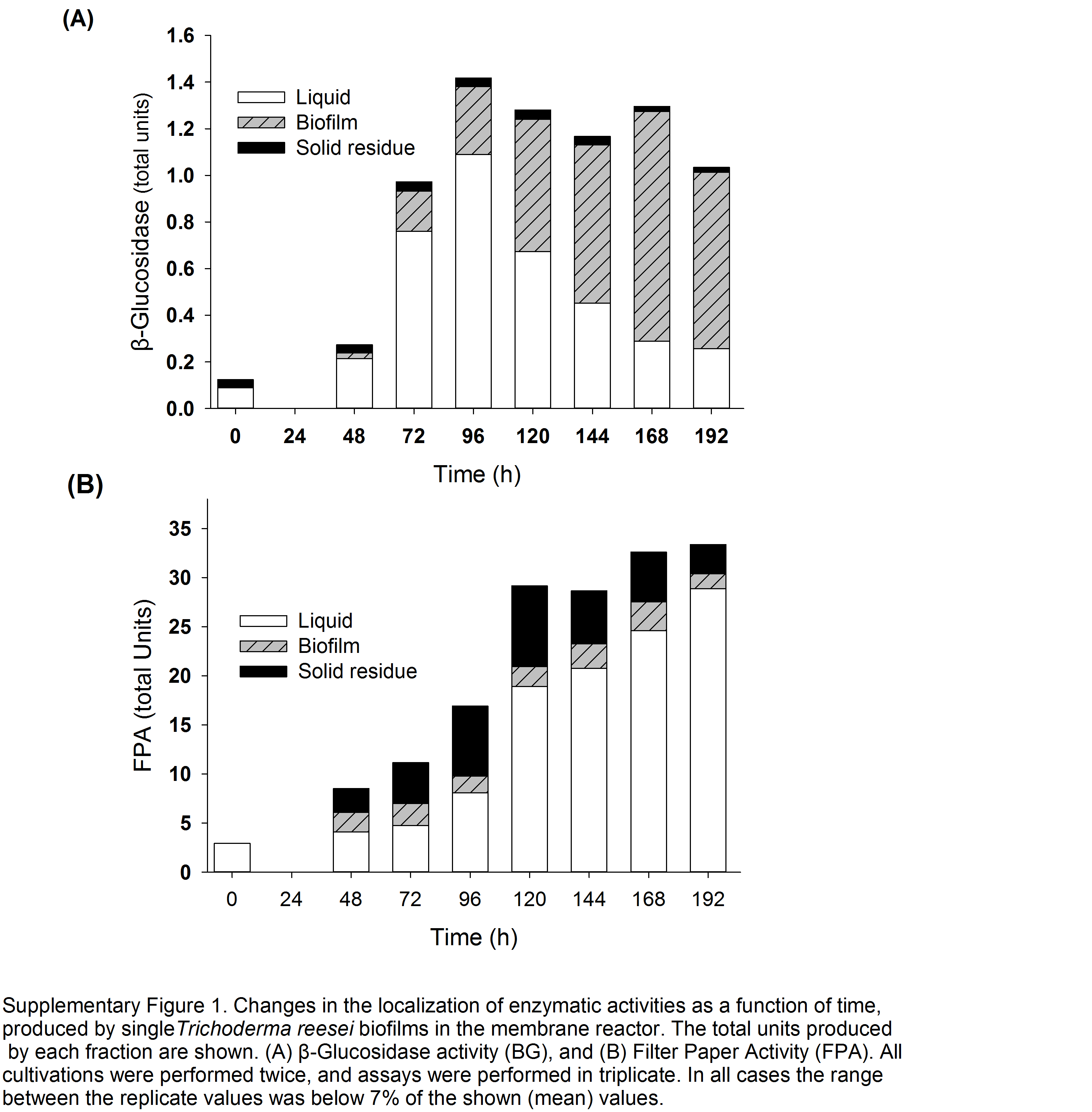

Supplement: Supplementary file 1 [file Image1.TIF]

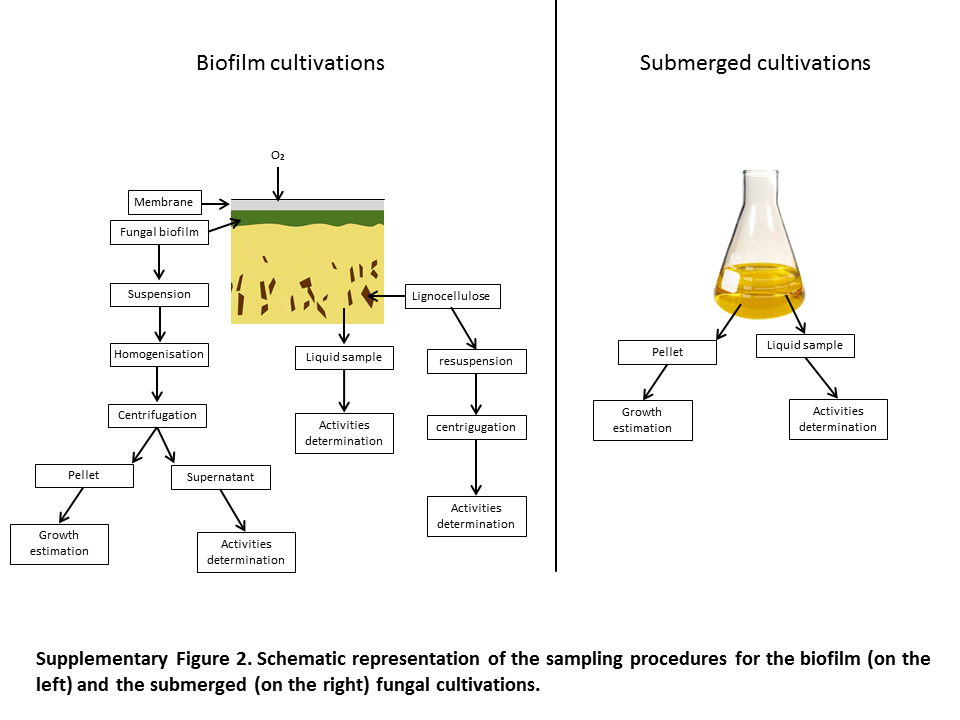

Supplement: Supplementary file 2 [file Image2.TIF]
